# Transplant Trial Watch

**DOI:** 10.3389/ti.2023.12039

**Published:** 2023-10-24

**Authors:** Simon R. Knight, John Fallon, Keno Mentor

**Affiliations:** ^1^ Oxford Transplant Centre, Churchill Hospital, Oxford, United Kingdom; ^2^ Centre for Evidence in Transplantation, Nuffield Department of Surgical Sciences, University of Oxford, Oxford, United Kingdom; ^3^ University Hospitals Coventry and Warwickshire NHS Trust, Coventry, United Kingdom

**Keywords:** delayed graft function, deceased donor kidney transplantation, randomised controlled trial, liver transplant, normothermic machine perfusion

To keep the transplantation community informed about recently published level 1 evidence in organ transplantation ESOT and the Centre for Evidence in Transplantation have developed the Transplant Trial Watch. The Transplant Trial Watch is a monthly overview of 10 new randomised controlled trials (RCTs) and systematic reviews. This page of Transplant International offers commentaries on methodological issues and clinical implications on two articles of particular interest from the CET Transplant Trial Watch monthly selection. For all high quality evidence in solid organ transplantation, visit the Transplant Library: www.transplantlibrary.com.

RANDOMISED CONTROLLED TRIAL 1 Balanced Crystalloid Solution Versus Saline in Deceased Donor Kidney Transplantation (BEST-Fluids): A Pragmatic, Double-Blind, Randomised, Controlled Trial.

*by Collins, M. G., et al. Lancet 2023; 402(10396): 105–117.*

## Aims

To assess if use of balanced crystalloid vs. saline reduces rates of delayed graft function.

## Interventions

The intervention group received a balanced crystalloid in the form of Plasma-Lyte 148 intra- and post-operatively for intravenous volume replacement vs. standard care who received 0.9% sodium chloride.

## Participants

808 participants, adults and children receiving kidney only transplant.

## Outcomes

The primary outcome was DGF, which they defined as need for dialysis within the first 7 days. Secondary outcomes included: number of dialysis treatments, duration of dialysis in days, ranked composite of DGF and day 2 creatinine reduction ratio, post-op hyperkalaemia, peak potassium, fluid overload, urine output, use of inotropes, acute rejection, number of biopsies, mortality, graft survival, graft function and hospital stay.

## Follow-Up

52 weeks.

## CET Conclusion

This large, multi-centre, double-blinded, randomised control trial found a reduction in DGF rate with the use of balanced crystalloid (30%) compared with normal saline (40%), a RR of 0.74 (*p* < 0.0001). The trial was well designed, with exceptional blinding, the Plasma-Lyte and saline were packaged in custom identical bags only identifiable by serial numbers, so all parties were blinded for the duration of the trial. The trial was conducted across 16 sites, with a representative trial cohort of deceased donor kidneys (DBD:DCD of 3:1), containing only 16 (2%) of pre-emptive recipients and 20 (2%) kidneys that received HMP as preservation, which is crucial given their primary outcome. This reduction in DGF equated to 190 fewer dialysis sessions in the balanced crystalloid group and a number needed to treat of 10 to prevent 1 case of DGF. Their hypothesised effect of the fluid on post-transplant biochemistry, with reduced chloride burden, increase bicarbonate and pH with minimal effect on potassium was demonstrated and thus reduced tubular acidosis and improve blood flow leading to lower rates of DGF is sound. The trial has few limitations, laboratory data was not collected beyond post-operative day 2 and other minor data points such as blood pressure and surgical anastomosis time, however given the trial size and randomisation strategy limiting centre effect, this is likely of no consequence. It is important to note that the effect is not necessarily generalisable to other balanced crystalloids such as Hartman’s, given that contains more chloride as well as lactate and further work would be needed to assess their benefit. This trial provides robust evidence sufficient to warrant consideration of changing practice, Plasma-Lyte is readily available, relatively inexpensive and in the context of renal transplant providing likely reduction in DGF.

## Jadad Score

5.

## Data Analysis

Modified intention-to-treat analysis.

## Allocation Concealment

Yes.

## Trial Registration

ACTRN12617000358347; ClinicalTrials.gov—NCT03829488.

## Funding Source

Non-industry funded.

RANDOMISED CONTROLLED TRIAL 2 Normothermic Machine Perfusion of Donor Livers for Transplantation in the United States—A Randomized Controlled Trial.

*by Chapman, W. C., et al. Annals of Surgery 2023 [record in progress].*

## Aims

The aim of this study was to investigate the effectiveness of normothermic machine preservation (NMP) versus static cold storage (SCS) in the prevention of preservation-related graft injury.

## Interventions

Donor livers were randomised to undergo either NMP or SCS.

## Participants

383 donor livers were randomised out of which 266 donor livers were transplanted.

## Outcomes

The primary endpoint was early allograft dysfunction (EAD). Secondary endpoints included graft survival, patient survival, incidence of postreperfusion syndrome, biochemical liver function, biliary complications, histological evidence of ischemia-reperfusion injury, feasibility and safety, health economics and organ utilization.

## Follow-Up

12 months.

## CET Conclusion

This unblinded randomised trial compared the outcomes of liver transplantation following either normothermic machine perfusion (NMP) or static cold storage (SCS). The study employed a “device-to-donor” methodology where the Organox metra device is transported to the site of organ retrieval, which the authors highlight is logistically more challenging. 266 livers were included in the analysis. The primary endpoint was early allograft dysfunction (EAD), defined as abnormal liver parameters 7 days after transplantation. There was no significant difference in EAD between the two groups. Although the difference in EAD was numerically greater when using an as treated or sub-group analysis of higher risk groups (high DRI, DCD donor), this to failed to reach statistical significance. The authors reached conclusions similar to that of previous European trials—NMP is a safe modality and shows potential to improve outcomes in marginal organs.

## Jadad Score

3.

## Data Analysis

Strict intention-to-treat analysis.

## Allocation Concealment

Yes.

## Trial Registration

ClinicalTrials.gov—NCT02478151.

## Funding Source

Industry funded.

## Clinical Impact Summary

The use of machine preservation technologies in liver transplantation has been gaining pace over recent years, with centres using a mixture of normothermic machine perfusion (NMP), hypothermic oxygenated machine perfusion (HOPE) and normothermic regional perfusion (NRP). Machine preservation has the potential to resuscitate the liver, reverse retrieval-related injury, allow longer safe preservation times and enable viability assessment prior to implant. In particular, NMP allows functional assessment of the liver with well-defined parameters predicting early allograft function [1].

The first multicentre randomised controlled trial (RCT) of normothermic machine perfusion in Europe was published in 2018, and demonstrated a significant (50%) reduction in the incidence of early allograft dysfunction (EAD) in machine perfused livers, despite longer preservation times [2]. These results were replicated in a US study (using a different NMP device), which also demonstrated a significant reduction in the incidence of EAD with NMP [3]. Whilst not specifically designed to demonstrate differences in organ utilisation, both studies also showed a reduction in organ discard rates, particularly for donation after cardiac death (DCD) livers.

In a recent publication in the Annals of Surgery, Chapman et al. report the results of the large multicentre US experience of NMP [4]. They used a protocol very similar to that followed in the European RCT. Livers were randomised to either conventional static cold storage (SCS) or NMP, with perfusion initiated at the donor hospital and the liver transported on the device to the implanting centre. In contrast to the European study, the trial did not meet its primary endpoint of demonstrating an overall reduction in EAD. Per-protocol analysis showed similar trends to the prior European and US studies, with greater reduction in EAD rates seen with NMP in DCD and high donor-risk index (DRI) subgroups. Interestingly, there was evidence of a learning curve, with a reduction in EAD rates in the NMP arm following enhanced training during the study. Unlike the previous two RCTs, there was no difference in transplant rate between the arms.

One important point to note is that all three RCTs used NMP in a “device-to-donor” configuration, with initiation of NMP at the donor hospital and transport on the device. This has significant logistical challenges, particularly in countries like the US where travel distances are longer and travel by plane is more common. In reality, most centres using NMP routinely in the UK and Europe are using NMP in a “back-to-base” configuration, with transport of the liver under SCS and initiation of perfusion in the recipient centre. Whilst small studies suggest that this does not compromise outcomes for the majority of livers [5], there is no large-scale RCT evidence to support the back-to-base NMP perfusion strategy that many centres are employing.

Overall, whilst this study demonstrates a smaller effect size than previous RCTs, it does confirm that the technology is safe and that the main benefit of this technology appears to be for more marginal (high DRI and DCD) livers.

### Clinical Impact

3/5.
